# Glial cell changes in the corpus callosum in chronically-starved mice

**DOI:** 10.1186/s40337-023-00948-z

**Published:** 2023-12-18

**Authors:** Annelie Zimmermann, Natalie Böge, Katharina Schuster, Anna Staffeld, Stephan Lang, Sadaf Gill, Hanna Rupprecht, Linda Frintrop

**Affiliations:** Institute of Anatomy, Rostock University Medical Center, Gertrudenstr. 9, 18057 Rostock, Germany

**Keywords:** Anorexia nervosa, Semi-starvation induced hyperactivity, Astrocytes, Microglia cells, Oligodendrocytes

## Abstract

Anorexia nervosa (AN) is characterized by emaciation, hyperactivity, and amenorrhea. Imaging studies in AN patients have revealed reductions in grey and white matter volume, which correlate with the severity of neuropsychological deficits. However, the cellular basis for the observed brain atrophy is poorly understood. Although distinct hypothalamic centers, including the arcuate nucleus (ARC) are critically involved in regulating feeding behavior, little is known about potential hypothalamic modifications in this disorder. Since glia e.g. astrocytes and microglia influence neuronal circuits, we investigated the glial changes underlying pathophysiology of starvation in the corpus callosum (CC) and hypothalamus. Female mice were given a limited amount of food once a day and had unlimited access to a running wheel until a 20% weight reduction was achieved (acute starvation). This weight reduction was maintained for two weeks to mimic chronic starvation. Immunohistochemistry was used to quantify the density of astrocytes, microglia, oligodendrocytes, and the staining intensity of neuropeptide Y (NPY), a potent orexigenic peptide. Chronic starvation induced a decreased density of OLIG2^+^ oligodendrocytes, GFAP^+^ astrocytes, and IBA1^+^ microglia in the CC. However, the densities of glial cells remained unchanged in the ARC following starvation. Additionally, the staining intensity of NPY increased after both acute and chronic starvation, indicating an increased orexigenic signaling. Chronic starvation induced glial cell changes in the CC in a mouse model of AN suggesting that glia pathophysiology may play a role in the disease.

**Supplementary Information:**

The online version contains supplementary material available at 10.1186/s40337-023-00948-z.

## Abstract (plain language summary)

The eating disorder anorexia nervosa (AN) leads to extreme body weight loss, increased physical activity, and the absence of menstrual periods. Studies have revealed reduced brain volumes in patients with AN, which are associated with the severity of cognitive impairments. The cellular basis for this brain volume loss is mostly unclear. Glial cells, recognized for their role as supporting tissue for neuronal cells, may be involved as they can influence neuronal mechanisms. Although distinct brain regions, like the hypothalamus, are critically involved in regulating feeding behavior, little is known about cell changes in that brain region of patients with AN. To investigate these changes, an animal model mimicking the symptoms of AN was used. Glial cell changes in the corpus callosum, which connects the two hemispheres of the brain, were observed. Furthermore, no glial cell changes in the arcuate nucleus of the hypothalamus were obtained. The findings indicate that glial cell changes in the corpus callosum may play a role in the disease.

**Supplementary Information:**

The online version contains supplementary material available at 10.1186/s40337-023-00948-z.

## Introduction

Severe body weight loss, hyperactivity, and amenorrhea are characteristics of the psychiatric eating disorder anorexia nervosa (AN) [1]. Severe reductions in grey and white matter volume present in AN patients are associated with neuropsychological deficits such as learning impairments [2, 3]. In addition, changes in both the volume and microarchitecture of white matter in the corpus callosum (CC) have been demonstrated in patients with AN [4, 5]. New targets for medical interventions may be found within the complex neuronal processes that control eating behavior via hypothalamic circuits. Within these circuits, neuropeptide Y (NPY) and agouti-related peptide (AgRP)-expressing neurons in the arcuate nucleus (ARC) promote food intake, while pro-opiomelanocortin (POMC)-expressing neurons promote satiety. POMC is a preprohormone that is cleaved into bioactive peptides, such as α-melanocyte-stimulating hormones (α-MSH), which inhibit feeding by binding to melanocortin receptors [6, 7]. These orexigenic and anorexigenic signals are transmitted to other neuroanatomical regions of the hypothalamus, such as the paraventricular nucleus, lateral hypothalamic area (LHA), and extra-hypothalamic brain regions, to regulate food intake. Recent research has shown that glial cells, particularly astrocytes and microglia, play a role in modifying this mechanism of food intake (as reviewed in [8, 9]).

To investigate the cellular basis of starvation-induced changes in the brain, we used a semi-starvation-induced hyperactivity (SIH) mouse model involving a limited amount of food and access to running wheels [10, 11]. In this model, mice received reduced food intake until a 20% weight reduction was reached (one-week phase defined as acute starvation) and maintained for two weeks (chronic starvation). We have previously reported GFAP^+^ astrocyte density reductions in the CC and cerebral cortex in this model [12, 13]. These glial cell changes were accompanied by volume reductions in the same regions which are in line with the findings in humans with AN. Other studies also reported reductions of GFAP^+^ astrocyte density in the CC, prefrontal cortex, and hippocampus in a dehydration-induced anorexia (DIA) rat model [14, 15]. For microglia, an increased density in the hippocampus and prefrontal cortex was found in this anorexia model indicating microgliosis [16, 17]. Food intake-related pathways may be involved in starvation, as evidenced by the upregulation of *Npy* and *Agrp* mRNA expression in an activity-based anorexia (ABA) model which includes a limited time for feeding [18–20]. However, the results for *Pomc* mRNA expression have been conflicting, as two studies demonstrated an increase in *Pomc* expression in the arcuate nucleus (ARC) in ABA animals, whereas one study reported a decrease in *Pomc* expression [18, 21, 22]. In addition, the POMC^+^ cell densities in the ARC in ABA animals were increased [23]. Despite these reports, the reasons for changes in glial density due to starvation are unclear (reviewed in [24]).

Here, we investigate volume and glial changes in the CC in a murine SIH model. Additionally, we investigate whether starvation leads to glial cell changes in the ARC and LHA, which may be associated with changes in NPY and POMC intensities.

## Material and methods

### Animals

Female C57BL/6J mice (n = 60 animals, 4-weeks old referred to as early adolescents and 8 weeks-old referred to adolescents) were purchased from Janvier Labs (Le Genest-Saint-Isle, France) as part of a large cohort study [11, 25] and housed under a 12/12 h light/dark cycle and a temperature of 22 ± 2 °C. The beginning of the light phase was at 6 AM. Female mice were used since a higher prevalence of AN in females is observed in humans [24]. All mice were housed individually in a cage and had unlimited access to a running wheel for the whole experiment. Once a week the mice are transferred to a new cage with fresh bedding and a fresh water bottle, and microbiological analysis was conducted by the recommendations of the Federation of European Laboratory Animal Science Associations (FELASA). The experiments were approved by the Review Boards for the Care of Animal Subjects of the district government of Mecklenburg-Western Pomerania (Reference Number 7221.3-1-005/21).

### Study design

As previously described, acute and chronic starvation was induced with the SIH model (previously also called modified activity-based anorexia (ABA) model) [10]. A phase of ten days with ad libitum access to food and water was used for the acclimatization of the mice. At 1 PM daily, we monitored the mice including measurements of body weight, food consumption, menstrual cycle, and performing of feeding.

In the ten days acclimatization phase, the mice received food ad libitum. The average daily food intake per mouse was calculated by averaging the consumed food in the acclimatization phase. Acute starvation was defined as one-week long phase in which the mice received 40% of the average daily food intake until a 20% reduction in body weight was obtained (Control_acute: n = 10; SIH_acute: n = 20). Chronic starvation was induced by two weeks of starvation following the acute starvation phase (Control_chronic: n = 10; SIH_chronic: n = 20). The food intake was adjusted daily to maintain the body weight loss, ranging between 45 and 70% of the average daily food intake. In each starvation phase, the mice were allowed free access to their calculated amount of food for the following 24 h. Throughout the various phases of starvation, the control group animals consistently had unrestricted access to food. To investigate running activity (previously described [11]), we utilized running wheels (11.5 cm in diameter) attached to the top of the cages. The running distance was evaluated every hour using an activity program (VitalView Activity 1.4, STARR Life Science Corp.).

### Volume measurements

Mice were injected intraperitoneally (i.p.) with ketamine (100 mg/kg) and xylazine (10 mg/kg). After transcardial perfusion with phosphate-buffered saline (PBS) and 3.7% paraformaldehyde solution (pH 7.4), the brains were rapidly dissected. The brains were post-fixed with a paraformaldehyde solution for 1 day, rinsed in tap water, and cryo-protected by immersion overnight in 10%, 20%, and 30% sucrose in PBS at 4 °C. The brains were embedded in optimal cutting temperature medium (Sakura, USA) and stored at − 20 °C. The entire brain of each mouse was cut frontally in a series of 40-µm sections on a cryostat (Leica CM 3050S, Nussloch, Germany) and every third section was thaw-mounted on glass slides for volume measurements. These sections were stained with Nissl staining following standard protocols. The other sections were incubated in a cryoprotection solution at − 20 °C and used for immunohistochemistry.

Nissl-stained sections were digitalized and the region of interest (ROI), the CC, of every second section were determined manually by tracing with ImageJ software (1.48v, Wayne Rasband, National Institutes of Health, Bethesda, MD, USA), by an observer blinded to the experimental treatment groups. Using the Cavalieri method, the areas were multiplied by the distance between the histological sections and summed to yield the total volume. The CC volume was analyzed from Bregma 1.85–1.46 using the Allen mouse brain atlas, encompassing the compartment completely. Due to insufficient slide quality, 4 animals were excluded from the analysis.

### Histology, immunohistochemistry, and image analysis

As previously described, immunohistochemistry was performed with standard procedures [12, 26, 27]. First, the sections were exposed to 5% goat, rabbit, or horse serum (Sigma, Munich, Germany) for 90 min and incubated overnight at 4 °C to the primary antibodies listed in Table 1.

**Table 1:** List of primary antibodies

Primary antibodies	Species	Dilution	Clonality	Purchase number	RRID	Supplier
GFAP	Goat	1:500	Polyclonal	SAB2500462-100UG	AB_10603437	Sigma-Aldrich, Germany
IBA1	Rabbit	1:1000	Polyclonal	019–19741	AB_839504	Wako, Japan
OLIG2	Rabbit	1:500	Polyclonal	AB9610	AB_570666	Millipore, Germany
NPY	Rabbit	1:4000	Polyclonal	ab30914	AB_1566510	Abcam, UK
POMC	Rabbit	1:250	Monoclonal	ab254257	–	Abcam, UK

Afterward, the sections were treated with 0.35% hydrogen peroxide (H_2_O_2_) in PBS for 30 min. Then, the sections were exposed to the corresponding secondary antibodies: rabbit anti-goat antibody (1:250, polyclonal rabbit antibody, purchase number: BA-5000, Vector Laboratories, Burlingame, CA, USA) or goat anti-rabbit antibody (1:250, goat polyclonal antibody, purchase number: BA-1000, Vector Laboratories). This step was followed by the ABC complex (Vector Laboratories, Burlingame, CA, USA). Finally, the detection of antigenic sites was performed by a reaction with 3,3’-diaminobenzidine (Dako, Hamburg, Germany). Negative controls (omission of the primary antibodies) were used. The slides were digitalized with the Leica DM6 B microscope equipped with a DMC6200 camera (Leica Microsystems CMS GmbH Wetzlar, Germany; 20-fold objective, numerical aperture (NA): 0.55; 40-fold objective, NA: 0.95).

For the GFAP staining, the sections at Bregma − 1.12 mm were used, while for the IBA1 and OLIG2 staining, the sections at Bregma − 1.54 mm were used. In each investigation, two immunohistochemically stained sections were digitized, and the results were averaged. For the quantification of the number of GFAP^+^, IBA1^+^, and OLIG2^+^ cells, the software ViewPoint (version 1.0.0.9628, PreciPoint, Freising, Germany) was used. The cells were counted by two evaluators blinded to the treatment group. Results are presented as cells per mm^2^. The observers counted all GFAP^+^, IBA1^+^, and OLIG2^+^ cells containing a visible nucleus. The CC was measured in three regions (next to the midline, under the subcingulum, and laterally) and the results were averaged. We excluded sections when the ROI was damaged.

In terms of ARC localization, for the GFAP staining, the sections at Bregma − 1.34 mm were used, while for the IBA1 and OLIG2 staining, the sections at Bregma − 1.46 mm were used. The ARC located near the third ventricle was reconstructed using the Allen Mouse Brain Atlas and the continuous Nissl staining of every third brain slice (Additional file 1: Fig. S1A). Following this orientation, the reconstruction of the ARC was performed manually for every section. The second ROI the LHA was defined as the area between the fornix (f) and optic nerve. Therefore, one perpendicular line was dropped between the most parietal part of the fornix and the most parietal part of the optic nerve. The other perpendicular line was dropped between the basal part of the fornix and the lowest visible basal part of the optic nerve.

To measure the staining intensity of the POMC and NPY sections, the software ImageJ (version 1.53t NIH, Bethesda, MD, USA) was used. For this analysis, the images were converted into grayscale images, and the pixels were divided into black or white by using a threshold. Results are presented as % stained area.

### Statistics

Data are displayed as mean and standard error of the mean (SEM). To determine the minimum group size required to significantly differentiate between controls and SIH mice regarding cell density changes, an *a-priori* one-way ANOVA power analysis was calculated using 80% power and a 5% alpha error with the software G*Power [28]. Differences between groups were statistically tested using the software GraphPad Prism (version 8.0.0, GraphPad Software, San Diego, CA, USA) with confidence intervals of 0.05. *p*-values of ≤ 0.05 were considered to be statistically significant. After the Grubbs test the datasets were analyzed via the Shapiro-Wilks tests for normal distribution. The parameters of CC volume, cell densities of GFAP^+^, IBA1^+^, and OLIG2^+^, as well as NPY and POMC staining intensities, were analyzed by students t-tests. When data was not normally distributed, the Mann–Whitney test was used. The following symbols are used to indicate the level of significance: *p ≤ 0.05, **p ≤ 0.01, ***p ≤ 0.001.

## Results

### Chronic starvation induces reductions in GFAP^+^, IBA1^+^, and OLIG2^+^ cell numbers in the CC

**Fig. 1:**
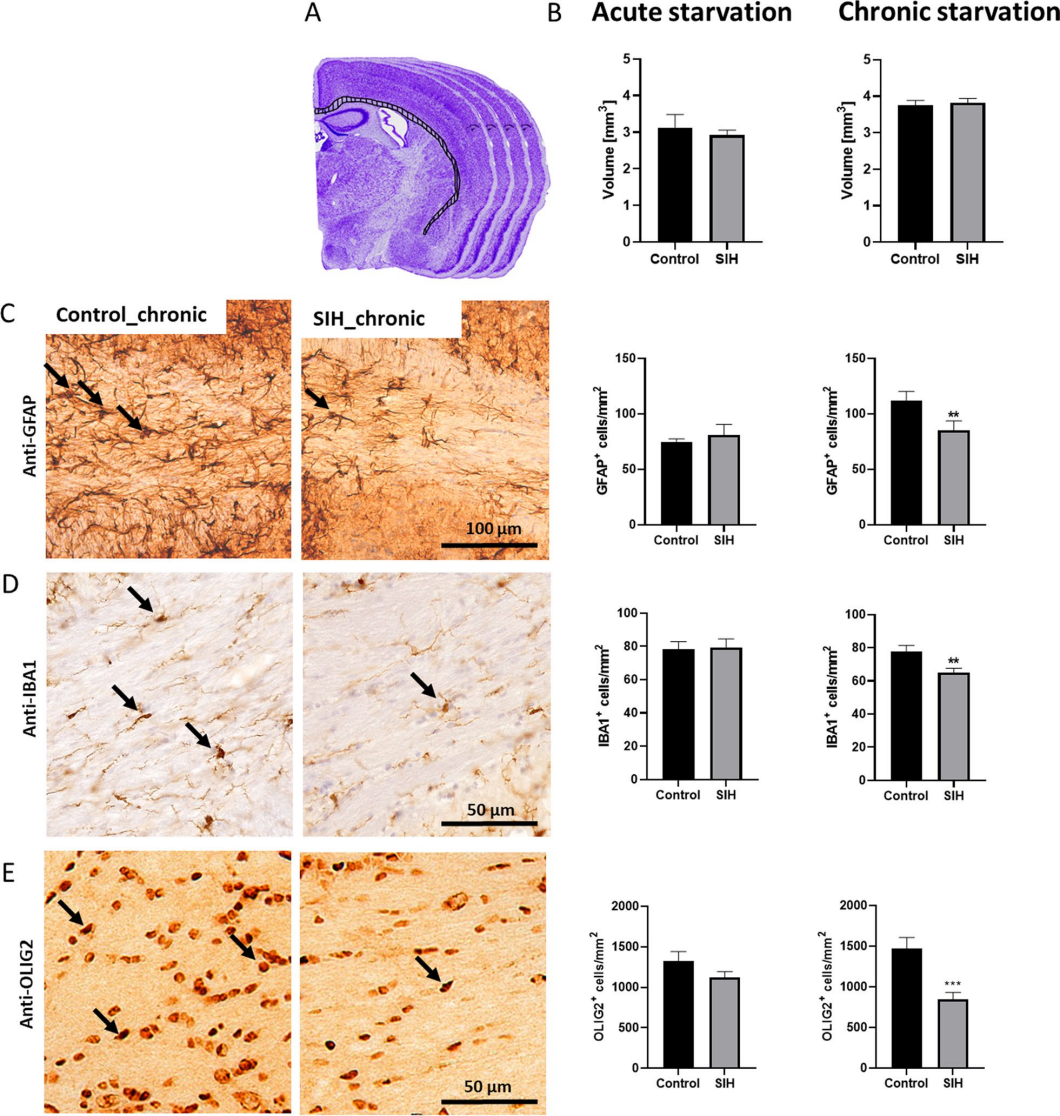
Chronic starvation leads to decreased astrocyte, microglia, and oligodendrocyte cell density in the corpus callosum. (**A**, **B**) The volumes of the corpus callosum were not changed in the SIH group compared to the controls. Cell numbers of (**C**) GFAP^+^ astrocytes, (**D**) IBA1^+^ microglia, and (**E**) OLIG2^+^ oligodendrocytes in the corpus callosum of SIH and control mice after acute and chronic starvation. The arrows mark (**C**) GFAP^+^, (**D**) IBA1^+^, and (**E**) OLIG2^+^ cells. After chronic starvation, the astrocyte, microglia, and oligodendrocyte cell numbers in SIH mice were significantly reduced in the corpus callosum. Two-sided Student’s t-test for acute starvation, Mann–Whitney test for chronic starvation, **p ≤ 0.01, ***p ≤ 0.001

Acute and chronic starvation did not lead to a change in CC volume in SIH mice in comparison to the corresponding control (Fig. 1B).

Next, we analyzed the influence of starvation on glial cells in the CC (Fig. 1C–E). Acute starvation did not lead to a change in GFAP^+^ cells in the CC. In contrast, chronic starvation induced a decrease in GFAP^+^ cells in this region (Control_chronic: 111.73 cells/mm^2^ ± 8.48 vs. SIH_chronic: 85.04 ± 6.8, *p* = 0.01, Fig. 1C). Further, acute starvation did not lead to a change in IBA1^+^ microglia cell density in the CC, whereas chronic starvation induced a decrease in this cell density (Control_chronic: 77.82 cells/mm^2^ ± 3.61 vs. SIH_chronic: 65.01 ± 2.6, *p* = 0.01, Fig. 1D). In parallel, acute starvation did not lead to changes in OLIG2^+^ cell number. In comparison, chronic starvation induced a reduction of OLIG2^+^ cells (Control_chronic: 1471.16 cells/mm^2^ ± 138.7 vs. SIH_chronic: 850.36 ± 82.84, *p* = 0.0003, Fig. 1E). In summary, chronic starvation reduced the numbers of GFAP^+^, IBA1^+^, and OLIG2^+^ cells in the CC.

### Starvation does not lead to glial changes in the ARC

**Fig. 2:**
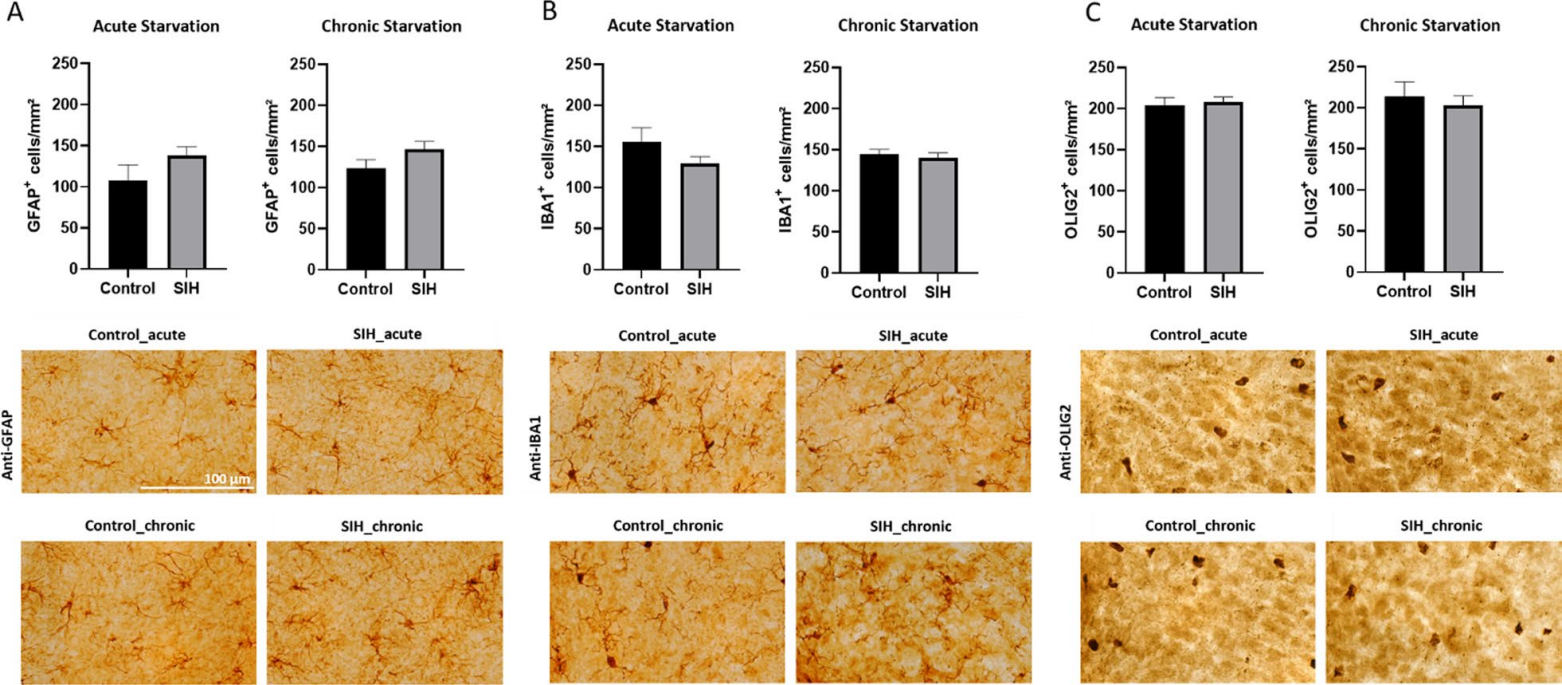
Starvation does not induce glial changes in the arcuate nucleus. Cell densities of (**A**) GFAP^+^, (**B**) IBA1^+^, and (**C**) OLIG2^+^ in the arcuate nucleus of SIH and control mice after acute and chronic starvation. Scale bar = 100 µm. Two-sided Student’s t-test

Next, we analyzed whether starvation altered glial cell numbers in the ARC in the hypothalamus (Fig. 2).

In the ARC, starvation did not change the cell densities of GFAP^+^ astrocytes and IBA1^+^ microglia. Furthermore, the OLIG2^+^ cell densities in the ARC were not altered in the SIH mice compared to control mice after acute and chronic starvation. In summary, the number of glial cells in the ARC did not change after starvation.

### Starvation does not lead to glial changes in the LHA

**Fig. 3:**
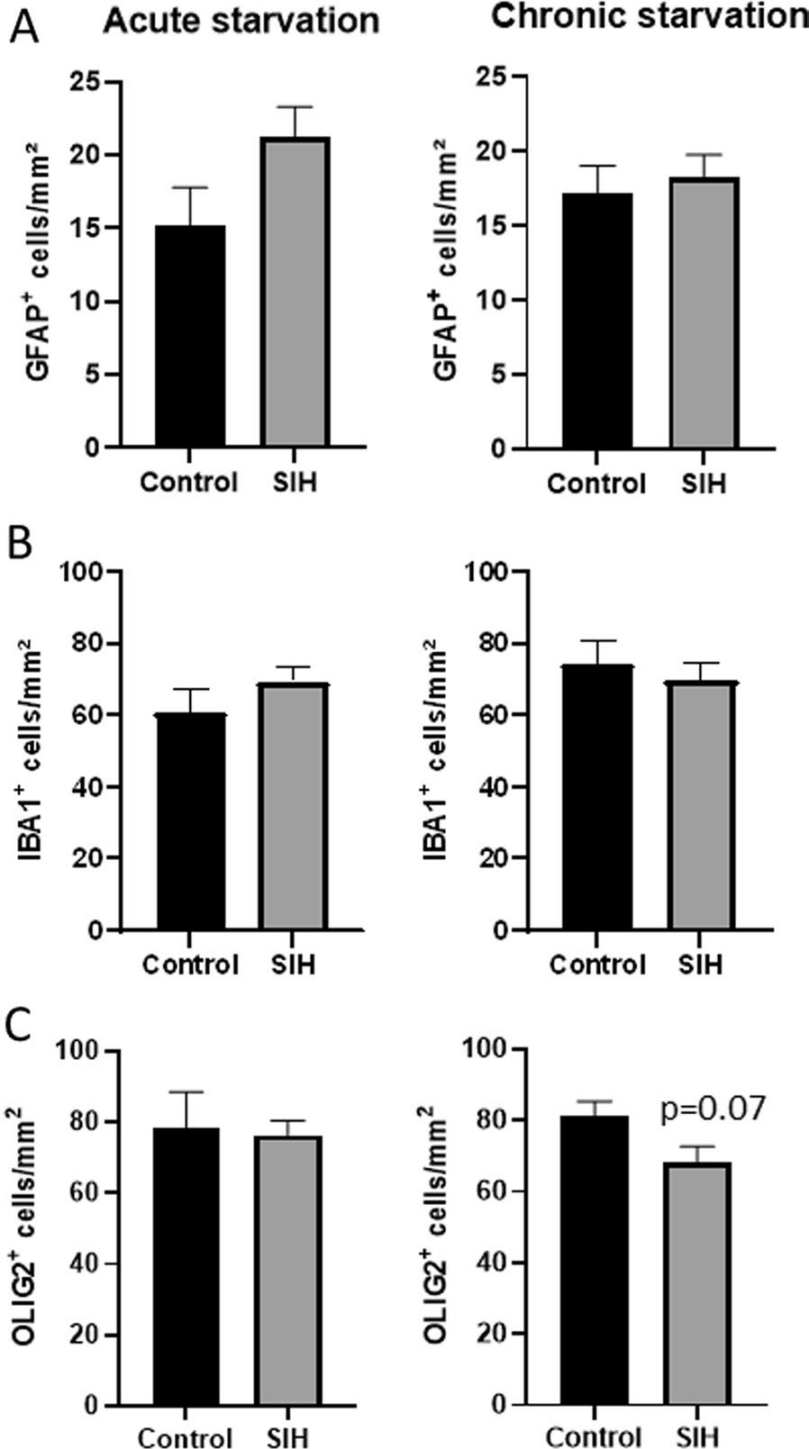
Starvation does not induce glial changes in the lateral hypothalamic area. Cell densities of (**A**) GFAP^+^, (**B**) IBA1^+^, and (**C**) OLIG2^+^ cells in the lateral hypothalamic area of SIH and control mice after acute and chronic starvation. Two-sided Student’s t-test

The ARC signals to the LHA which was chosen as a second region of interest because of its involvement in the regulation of food intake (Fig. 3).

In the LHA, the cell densities of GFAP^+^ astrocytes, and IBA1^+^ microglia were not altered either after acute starvation or after chronic starvation. Similarly, there was no difference in OLIG2^+^ cells between the control and SIH mice after acute starvation, while a trend to a lower number of OLIG2^+^ cells in SIH mice was observed after chronic starvation (Control_chronic: 81.03 cells/mm^2^ ± 4.25 vs. SIH_chronic: 67.97 cells/mm^2^ ± 4.69, *p* = 0.07, Fig. 3C). In summary, the number of glial cells in the LHA did not change after starvation.

### Starvation increases NPY expression in the ARC

**Fig. 4:**
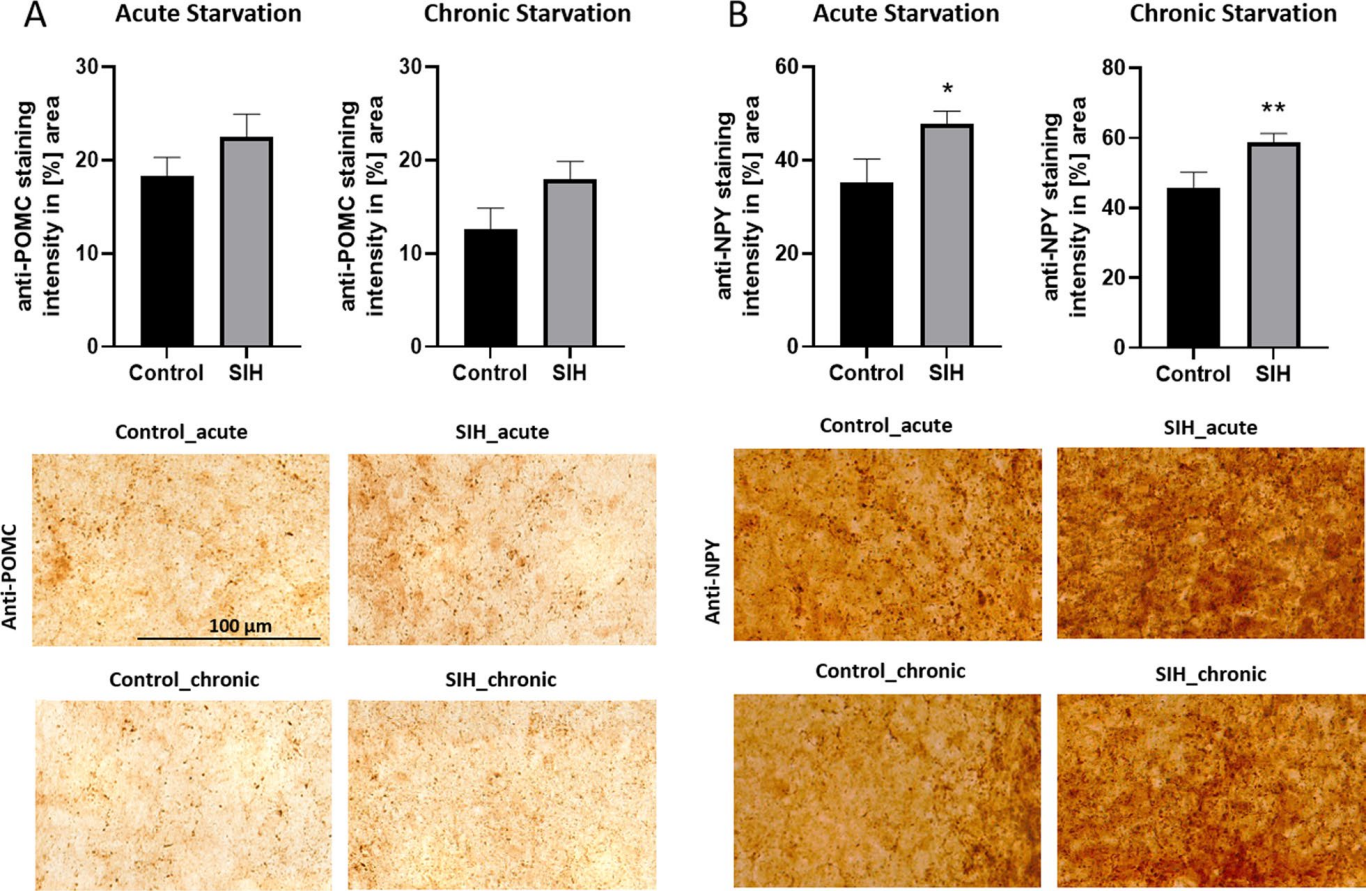
Acute and chronic starvation induces an increase in NPY staining intensity in the arcuate nucleus. Staining intensity of (**A**) POMC and (**B**) NPY in the arcuate nucleus (ARC) of SIH and control mice during acute and chronic starvation. Acute and chronic starvation led to an increase in NPY staining intensity. Scale bar = 100 µm. Two-sided Student’s t-test, *p ≤ 0.05, **p ≤ 0.01

Peptides NPY and POMC are involved in food intake regulation. After acute and chronic starvation, the expression of POMC in SIH mice did not change compared to controls (Fig. 4A).

In comparison, the staining intensity of NPY in SIH mice was increased after acute starvation (Control_acute: 35.32% stained area ± 4.97 vs. SIH_acute: 47.72% stained area ± 2.80, *p* = 0.04, Fig. 4B). The staining intensity of NPY was also increased after chronic starvation (Control_chronic: 45.89% stained area ± 4.34 vs. SIH_chronic: 58.86% stained area ± 2.41, *p* = 0.01, Fig. 4B).

## Discussion

To unravel the neuropathology of AN, a murine anorexia model, which leads to the AN-related core symptoms of hyperactivity, and amenorrhea, was used [11]. Our previous studies showed a reduction in CC volume in rats with a 25% weight reduction [12], whereas, no changes in SIH mice with a 20% weight reduction were observed suggesting the effects of white matter brain atrophy depending on the extent of starvation.

To examine astrocytopathy in an animal model of AN, we conducted a detailed analysis of the white matter, specifically focusing on the CC. Astrocytes fulfill various functions, i.e. involvement in building the blood–brain barrier, transporting nutrients, and gliotransmission which is an active information transfer from glial cells to neurons [8]. Furthermore, astrocytes actively regulate behavior and play an important role in behavioral disorders i.e. glial ablation in the prefrontal cortex of rats induced depressive-like symptoms [29]. In our study, the density of astrocytes in the CC remained unchanged after acute starvation, whereas it decreased following chronic starvation. Thus, changes in the density of astrocytes were dependent on starvation length indicating that prolonged periods of starvation may result in potential dysfunction of glial cells. These findings are consistent with previous studies in the rat SIH model, in which astrocyte reductions due to prolonged starvation were observed [12], and with those of studies of the DIA rat model [14, 15]. In the latter model, disruption of glutamate-glutamine homeostasis and a deramified morphology of astrocytes in the prefrontal cortex was found [30]. Thus, astrocyte reductions and their potential dysfunction may contribute to the neuropsychological deficits in human AN.

Next, we analyzed whether microglia, which are the resident immune cells of the brain, were influenced by starvation. The astrocyte changes paralleled with the finding that the density of IBA1^+^ cells in the CC decreased after chronic starvation. In contrast, in the DIA model, these cell densities were increased in the prefrontal cortex and hippocampus indicating a pro-inflammatory environment [16, 17]. Thus, we should further analyze the morphology of glial cells to estimate the extent of the microglia activation. Microglia cells may react differently depending on the type of starvation, the duration of the starvation, and the specific brain region affected.

As the primary cell type in the CC is the oligodendrocyte, which is responsible for both the myelination of axons and the metabolic support of neurons, we studied these cells in our model [31]. The density of OLIG2^+^ cells in the CC of SIH mice decreased after chronic starvation, suggesting the myelin status is altered due to starvation. The transcription factor Olig2 is expressed in oligodendrocytes and oligodendrocyte precursor cells (OPCs). In a previous study, we showed no alterations in the density of mature APC^+^ oligodendrocytes in SIH rats [12] suggesting that especially OPCs and premyelinating oligodendrocytes are affected by starvation. Additionally, hypoglycemia is known to induce apoptotic cell death in OPCs [32]. Thus, increased apoptosis may explain the reduced density of OLIG2^+^ cells. Notably, patients with AN have been found to exhibit decreased structural connectivity in the CC, whereas those undergoing body weight rehabilitation showed no change in this connectivity [5, 33]. In summary, starvation leads to glial cell changes in CC which were not associated with volume changes in this region.

The next region of interest was the hypothalamus, which functions to regulate hunger, satiety, energy metabolism, and body weight. We revealed that starvation did not lead to cell density changes in the ARC, which contains first-order neurons that regulate food intake. Similarly, no changes were observed in these cell densities in the LHA, where corresponding second-order neurons are located. Hence, our study suggests that the unchanged glial densities in the ARC and LH following starvation may imply a lack of glial involvement in these regions. Consequently, neuronal alterations and interactions between glial cells and neurons may be the primary focus for understanding starvation-induced changes. The absence of any alteration in microglia density suggests that there is no presence of microgliosis or inflammation in the ARC and LH.

In the current study, acute and chronic starvation increased NPY staining intensity in the ARC, whereas no changes were observed in the POMC intensities. Also, Rijke et al. demonstrated that the expression of neuropeptides in first-order neurons in the ARC is altered during negative energy states [18]. Therefore, it appears that starvation increases the orexigenic signaling, consistent with previous literature that reports an increase in *Npy* expression [18, 19]. Previous studies revealed conflicting results regarding the *Pomc* expression [18, 21, 22]. In ABA rats, there was an increase in the density of POMC^+^ cells in the ARC [23]. Thus, it appears that NPY is more generally affected by starvation, while POMC may be more vulnerable. Interestingly, inhibiting POMC neurons in ABA rats resulted in the inhibition of food anticipatory development, which is characterized by an increase in locomotor activity prior to the feeding period [34].

In conclusion, the SIH model indicated that glial cells in the CC play a role in the pathophysiology of eating disorders. Therefore, researching glial cells is important for finding new targets to treat patients with AN. In terms of that, we have to be aware of the limitation of the SIH model, as its results are exclusively based on animals. Nevertheless, the SIH model represents many somatic aspects of AN, such as body weight loss, amenorrhea, and hyperactivity, assuming it to be highly translationally significant. Considering that the effects of starvation resemble the secondary effects of AN, it is essential to emphasize that studying voluntary food restriction as a potential treatment target may also be relevant. In future studies, we will investigate the interactions (and dysfunctions) between glial cells and neurons in the CC. To differentiate between the impact of starvation and hyperactivity, we will include two additional groups: one control group without running wheels and one starvation group without running wheels. Moreover, in future studies, we will include refeeding of the SIH mice to investigate whether the observed changes can be reversed by refeeding.

### Supplementary Information

**Additional file 1: Fig. S1.** (**A**) The Allen Mouse Brain Atlas and continuous Nissl staining of every third brain slice were utilized to reconstruct the region in the images of the immunohistochemical stainings for the arcuate nucleus (ARC), which is located near the third ventricle (3 v). (**B**) The lateral hypothalamic area (LHA) was defined as the area between the fornix (f) and optic nerve by dropping two lines between these structures. Scale bar = 500 µm.

## Data Availability

Data may be made available upon sending requests to the correspondence.
