# Supplementary figures and images for: Glial cell changes in the corpus callosum in chronically-starved mice
Source: J Eat Disord. 2023 Dec 18;11:227. doi: 10.1186/s40337-023-00948-z (PMC10726510; doi:10.1186/s40337-023-00948-z)

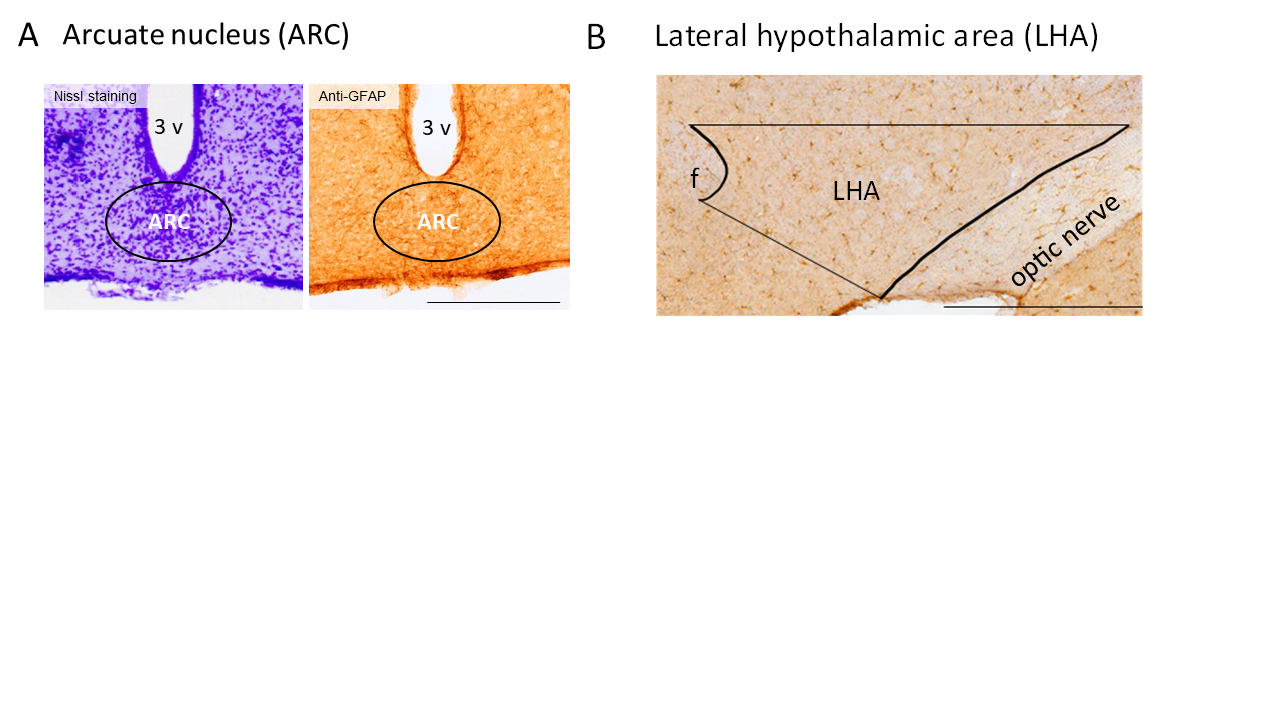

Supplement: Supplementary file 1 — Additional file 1: Fig. S1. (A) The Allen Mouse Brain Atlas and continuous Nissl staining of every third brain slice were utilized to reconstruct the region in the images of the immunohistochemical stainings for the arcuate nucleus (ARC), which is located near the third ventricle (3 v). (B) The lateral hypothalamic area (LHA) was defined as the area between the fornix (f) and optic nerve by dropping two lines between these structures. Scale bar = 500 µm. [file 40337_2023_948_MOESM1_ESM.tif]
